# Improved GMP compliant approach to manipulate lipoaspirates, to cryopreserve stromal vascular fraction, and to expand adipose stem cells in xeno-free media

**DOI:** 10.1186/s13287-018-0886-1

**Published:** 2018-05-11

**Authors:** Francesco Agostini, Francesca Maria Rossi, Donatella Aldinucci, Monica Battiston, Elisabetta Lombardi, Stefania Zanolin, Samuele Massarut, Pier Camillo Parodi, Alessandro Da Ponte, Giovanni Tessitori, Barbara Pivetta, Cristina Durante, Mario Mazzucato

**Affiliations:** 1 Stem Cell Unit, CRO Aviano National Cancer Institute, Aviano, PN Italy; 2 Clinical-Experimental Onco-Hematology Unit, CRO Aviano National Cancer Institute, Aviano, PN Italy; 3 Molecular Oncology Unit, CRO Aviano National Cancer Institute, Aviano, PN Italy; 4 Breast Surgery Unit; CRO Aviano National Cancer Institute, Aviano, PN Italy; 5 Department of Plastic and Reconstructive Surgery, University of Udine, Udine, Italy; 6 Cytogenetic Unit, AAS 5 Friuli Occidentale, “S. Maria degli Angeli” Hospital, Pordenone, Italy

**Keywords:** Stromal vascular fraction, Adipose tissue, Freezing protocol, Cell viability, CFU-F, Immunophenotype characterization, Adipose stem/stromal stem cells, Growth rate, Differentiation potential, Cell morphology, Karyotype, Anchorage independent growth, Good manufacturing practice, Advanced therapy medicinal product

## Abstract

**Background:**

The stromal vascular fraction (SVF) derived from adipose tissue contains adipose-derived stromal/stem cells (ASC) and can be used for regenerative applications. Thus, a validated protocol for SVF isolation, freezing, and thawing is required to manage product administration. To comply with Good Manufacturing Practice (GMP), fetal bovine serum (FBS), used to expand ASC in vitro, could be replaced by growth factors from platelet concentrates*.*

**Methods:**

Throughout each protocol, GMP-compliant reagents and devices were used. SVF cells were isolated from lipoaspirates by a standardized enzymatic protocol. Cells were cryopreserved in solutions containing different albumin or serum and dimethylsulfoxide (DMSO) concentrations. Before and after cryopreservation, we analyzed: cell viability (by Trypan blue); immunophenotype (by flow cytometry); colony-forming unit-fibroblast (CFU-F) formation; and differentiation potential. ASC, seeded at different densities, were expanded in presence of 10% FBS or 5% supernatant rich in growth factors (SRGF) from platelets. The differentiation potential and cell transformation grade were tested in expanded ASC.

**Results:**

We demonstrated that SVF can be obtained with a consistent yield (about 185 × 10^3^ cells/ml lipoaspirate) and viability (about 82%). Lipoaspirate manipulation after overnight storage at +4 °C reduced cell viability (−11.6%). The relative abundance of ASC (CD34^+^CD45^−^CD31^–^) and endothelial precursors (CD34^+^CD45^−^CD31^+^) in the SVF product was about 59% and 42%, respectively. A period of 2 months cryostorage in autologous serum with added DMSO minimally affected post-thaw SVF cell viability as well as clonogenic and differentiation potentials. Viability was negatively affected when SVF was frozen at a cell concentration below 1.3 × 10^6^ cells/ml. Cell viability was not significantly affected after a freezing period of 1 year.

Independent of seeding density, ASC cultured in 5% SRGF exhibited higher growth rates when compared with 10% FBS. ASC expanded in both media showed unaltered identity (by flow cytometry) and were exempt from genetic lesions. Both 5% SRGF- and 10% FBS-expanded ASC efficiently differentiated to adipocytes, osteocytes, and chondrocytes.

**Conclusions:**

This paper reports a GMP-compliant approach for freezing SVF cells isolated from adipose tissue by a standardized protocol. Moreover, an ASC expansion method in controlled culture conditions and without involvement of animal-derived additives was reported.

## Background

Adipose tissue, beside its role for energy storage, is a known source of stromal precursors and stem cells. These cells are enclosed in the so-called stromal vascular fraction (SVF), a heterogeneous population including hematopoietic cells, and adipose-derived stromal/stem cells (ASC). Approaches for the phenotypic characterization of SVF cells were previously suggested in position papers from the International Federation for Adipose Therapeutics and Science (IFATS) and from the International Society of Cellular Therapy (ISCT), as well as in other publications [1–3]. Such a composite and heterogeneous pool of cells can be used for clinical applications by virtue of the pro-angiogenetic and immune modulatory activity exerted by the different cell populations [1, 2]. SVF can be extracted from lipoaspirates by enzymatic digestion, followed by filtration and cell washing [4]. The possibility of storing purified SVF products by cryopreservation and freezing is crucial to optimize the study design for clinical applications in humans. Successful SVF cryopreservation using different protocols has been demonstrated in previously published works [3, 5] but there is not clear consensus about the appropriate freezing approach that maximally preserves cell viability.

In recent years, stromal cells have been utilized in a growing number of trials for different clinical applications [6]. SVF derived from adipose tissue is an abundant source of ASC [7, 8]. IFATS/ISCT proposed three minimal criteria for the definition of ASC: 1) plastic adherence; 2) expression of CD73, CD90, and CD105, and lack of expression of CD11b, CD14, CD19, CD45, and HLA-DR; and 3) differentiation potential into adipocytes, chondrocytes, and osteoblasts [9, 10]. Ex-vivo cell expansion must be performed to obtain a sufficient number of cells for potential use in humans; such “extensive manipulation” is subject to Advanced-Therapy Medicinal Product (ATMP) regulations [11]. ATMPs must be produced in compliance with current Good Manufacturing Practice (GMP) guidelines. As well as strict requirements concerning the production facilities (cell factories, personnel, procedures), GMP guidelines also regulate the quality of reagents and disposables used in the manufacturing process. Fetal bovine serum (FBS) is a well-known growth supplement for cell culture, but xeno-carbohydrates and xeno-proteins contained in FBS may lead to undesired clinical effects [12–15]. Thus, since 2001, replacement of FBS with suitable alternatives has been recommended for GMP-grade cell expansion throughout Europe and the United States [16]. The use of human growth factors derived from platelets for ex-vivo stem cell expansion is compliant with GMP guidelines [17–20]. In previous publications, growth factor release from platelets was obtained by repeated freeze and thaw cycles [18, 21–24]. In this work, we used a supernatant rich in growth factors (SRGF) produced, as previously published [25], through CaCl_2_ addition to the platelet apheretic product from healthy donors. SRGF final batches used in this work were manufactured by pooling together a number of single-donor products that has been shown to reduce the variability of growth factor concentrations [26]. The impact on mesenchymal stem cell proliferation and differentiation mediated by growth factors derived from platelets through CaCl_2_ addition has been previously investigated [17, 27, 28] but it is still not fully characterized. Furthermore, there is limited knowledge about the influence of SRGF on ASC physiology in cell culture. Noteworthy, in this paper, the SRGF-mediated effect on the ASC growth rate was assessed by seeding cells at different densities; the effect of plating density on cell physiology has been previously investigated only on mesenchymal stem cells derived from murine [29] or human [30–32] bone marrow. In this work, we aimed to define an efficient GMP-compliant method to cryopreserve SVF cells isolated from adipose tissue and to expand ASC using SRGF as medium additive, starting from a thawed SVF product. In particular, we investigated the impact of different cryopreserving solutions on viability, immunophenotype, clonogenicity, and differentiation capacity of nucleated cells (NC) or ASC enclosed in SVF. In addition, ASC were expanded at different cell seeding densities in 10% FBS- or 5% SRGF-containing media. We determined the impact of different culture conditions on the ASC expansion rate, immunophenotype, and differentiation potential. GMP-compliant materials, reagents, and devices were used.

## Methods

### Description of the study

This study obtained approval from the Ethics Committee of the CRO Aviano National Cancer Institute (protocol number: CRO-2016-30), and it was carried out in accordance with the Declaration of Helsinki (2004).

In this study, 19 leftover lipoaspirates were processed for SVF extraction, with 14 lipoaspirates being taken from the abdomen and the remaining samples from the thigh or hip/flank. The first six lipoaspirates were immediately treated after delivery to the cell-processing laboratory from the operating theatre, while the others were left at +4 °C for 16–18 h (overnight) before manipulation. Cell count and viability assay were performed immediately after SVF isolation. To optimize the protocol of such quality control test, the impact of performing red blood cell lysis before cell viability evaluation was tested in paired aliquots of the first four SVF products. Immunophenotypic analysis of SVF cells before cryopreservation was performed in nine SVF. Cryopreservation was performed using four different solutions. Due to limited availability of extracted SVF cells, simultaneous application of all four cryopreservation methods to each product was not possible; thus, solutions A and B were used in 10 out of 19 SVF, solution C in 9 out of 19 SVF, and solution D in 5 out of 19 SVF. The composition of each solution is described below. Cell viability was measured again in all SVF products upon thawing after 2 months of storage. Further investigations were performed on five SVF products cryopreserved by solutions C and D, in which we evaluated immunophenotype, colony forming unit-fibroblasts (CFU-F) growth, and differentiation potential. Moreover, three aliquots of these SVF products were used to assess viability upon thawing after 1 year of storage. The same five SVF samples that were thawed after 2 months of storage were placed in culture to extract and expand the ASC. We defined the impact of the cell seeding density on ASC growth rate in both 5% SRGF- or 10% FBS-containing media. Moreover, we tested the effect of the different media on ASC morphology, immunophenotype, differentiation potential, transformation, and karyotype stability.

### SVF isolation from adipose tissue

**Fig. 1:**
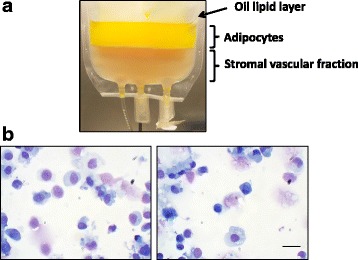
**a** Distinct stratified phases are clearly evident after 1 h of lipoaspirate treatment with collagenase solution at 37 °C. Stromal vascular fraction cells are contained in the lower clear phase; digested adipocytes as well as released oily lipids are stratified in the two upper phases. The image is representative of a completely accomplished digestion process. **b** Images of diluted stromal vascular fraction cells cytospun on a glass slide. Cells were differentially stained by May-Grunwald/Giemsa protocol. Scale bar = 10 μm

Lipoaspirates were derived from 19 female breast cancer patients (age 52.4 ± 1.6 years, body mass index 23.5 ± 2.4 kg/m^2^) who underwent quadrantectomy or total mastectomy and reconstructive lipotransfer. Fat tissue was harvested under local anesthesia by standard sterile liposuction techniques as described by Coleman [33, 34]. All samples were waste byproducts of surgery. SVF isolation was performed according to a previously published protocol [8] with modifications. Under a laminar flow biological cabinet, the lipoaspirate was transferred to a plastic bag (Transfer Bag, JMS Singapore PTE LTD, Singapore) and washed three times by adding warm (37 °C) clinical-grade Ringer Lactate solution (S.A.L.F. S.p.A. Laboratorio Farmacologico, Bergamo, Italy). After phase stratification, the lower phase was discarded. Thereafter, a solution of collagenase, 0.15 U/ml final concentration (NB 6 Good Manufacturing Practice grade, SERVA Electrophoresis GmbH, Heidelberg, Germany) was added to the washed lipoaspirate. The product was incubated for 60–70 min at 37 °C in a certified medical device (Plasmatherm Barkey GmbH & Co KG, Leopoldshoehe, Germany). The stratification (Fig. 1a) was considered to indicate complete lipoaspirate digestion. After collagenase neutralization by the addition of a human albumin solution (Albital 200 g/l, Kedrion S.p.A., Lucca, Italy) and product stratification, the lower phase was recovered and centrifuged at 400 × g for 10 min at +4 °C. The cell pellet was washed with a solution composed of 10% Albital, 10% Anticoagulant Acid Citrate Dextrose Solution–A (ACD-A; Haemonetics Corporation, Braintree, MA, USA), and 2 U/ml heparin (Epsodilave, HOSPIRA ITALIA S.r.l., Napoli, Italy) in Ringer Lactate solution.

### SVF cell count and viability analysis

Cell counts were performed using a Burker chamber. To estimate the total NC content, a small aliquot of the SVF final product was stained with 5% crystal violet dye (Sigma, St. Louis, MO, USA). SVF cell viability was estimated by Trypan blue dye exclusion test; an aliquot of the cell product was added to 0.4% Trypan blue dye (0.2% final concentration) (European Pharmacopeia 7.0, 2011). Only NC with an approximate circular shape were taken into account and, among these, only markedly colored cells were considered to be dead cells. In part of the samples, cell counts were performed by three independent and skilled operators to test the reliability of the performed evaluations; obtained data were correlated and consistent (data not shown).

Cell viability was calculated as follows: percent NC viability = viable NC / total NC × 100. For cell imaging purposes, an aliquot of SVF samples was diluted and cytospun by a standard protocol on a glass slide.

Elimination of red blood cells from the specimen can facilitate microscopic evaluation of NC. Red blood cell lysis cannot be performed on the cellular product administered to the patient since a GMP-grade lysis solution is not available: thus, features of the lysed biological specimen could be different from the product aimed to be administered to the patient. In this work, we tested the impact on NC viability results mediated by performing red blood cell lysis immediately before Trypan blue dye exclusion test. On a small aliquot of selected SVF products, lysis solution (PharmLyse, BD Biosciences, Becton-Dickinson, San Jose, CA) was added (5 min in wet ice) before cell staining with Trypan blue dye. In parallel, NC viability was evaluated in the same paired SVF products by Trypan blue dye staining, without the addition of lysis solution. In part of the SVF fresh samples, cells were cytospun and differentially stained by the May-Grunwald/Giemsa protocol. Images were taken by a digital color camera (Motic) (Fig. 1b).

### Immunophenotypic characterization of SVF and ASC by flow cytometry

After lysis of residual erythrocytes in PharmLyse solution (BD Biosciences), cells were washed, maintained in BD Pharmingen Stain Buffer, and labeled with a large panel of monoclonal antibodies (all from BD Biosciences): CD34 APC or PE-Cy7, CD45 APC-Cy7 or FITC, CD31 FITC, CD90 FITC or APC, CD73 PE, CD13 APC, CD44 FITC, CD29 APC, CD166 PE, CD10 PE or APC, HLA I/ABC PE, HLA II/DR FITC, CD106 PE, CD36 FITC, CD146 PE, CD235 (Glyα) PE, CD144 PE, CD11b APC, CD11c APC, CD14 APC, and CD105 PE (Beckman Coulter). Labeling was performed while keeping in each tube CD34 (clone 581), CD45, and 7-aminoactinomycin D (7-AAD; to discriminate dead cells). Glyα was used to confirm the exclusion of residual red blood cells from the analysis. In some cases, the SVF sample was labeled as received without lysis or washing, and Hoechst was added to the labeling combination in order to stain nuclei and confirm the gating strategy (data not shown).

Samples were acquired on a BD FACSCanto II flow cytometer and analyzed by BD Diva software. All experiments were performed after instrument calibration with CS&T beads (BD Biosciences). A multicolor gating strategy analysis was performed, excluding debris (using residual lymphocytes as an internal standard), and dead cells (based on 7-AAD), focusing on live intact cells [35]. CD34-positive (^+^) cells were gated and deeply characterized for expression of CD31 and subsequently of the other markers. Among the CD34-negative(^–^) cells, the CD45^+^ fraction was identified as residual white blood cells.

Aliquots of ASC, expanded at the seeding density of 1 × 10^3^ cells/cm^2^ in media with 10% FBS or 5% SRGF added for different cell passages (see below), were detached by trypsinization and washed in Pharmingen Stain Buffer. Cells were labeled with the same panel of monoclonal antibodies used for SVF.

### Cryopreservation and freezing of SVF

Cryopreservation was performed by resuspending SVF cell pellets in four different solutions. Solution A was prepared by adding the following to the standard saline solution (B. Braun, Melsungen, Germany): Albital (10%), ACD-A (5%), and dimethylsulfoxide (DMSO; 10%; CryoSure-DMSO, Li StarFish, Milan, Italy). Solution B was prepared by adding the following to the saline solution (B. Braun): human serum (50%), Albital (5%), ACD-A (2.5%), and DMSO (10%). Solution C and solution D were prepared by adding DMSO at 10% and 5%, respectively, to pure human serum. Thus, solutions A and B contained the saline solution, the same amounts of DMSO, and different concentrations of human serum, Albital, and ACD-A. The serum-based solutions C and D differed between each other only for the DMSO concentration, and they did not contain Albital or ACD-A. Solutions A, B, and C contained the same amounts of DMSO. Final concentrations were reported. Samples were frozen overnight at −80 °C in freezing containers designed to achieve a cooling rate of −1 °C/min (Mr Frosty, Thermo Scientific, Waltham, MA, USA). Afterwards, samples were stored in liquid nitrogen vapor phase (−190 °C).

### SVF thawing

SVF aliquots were thawed at 37 °C for 2 min and, to completely remove DMSO, cells were resuspended in 8 ml serum-free minimum essential medium eagle—alpha modification (α-MEM; Lonza, Basel, Switzerland) containing 100 IU/ml penicillin and 100 mg/ml streptomycin (both from Sigma) and were centrifuged at 260 × g for 5 min at room temperature. Serum-free medium was used to avoid the influence of any medium additive on the following cell assays or expansion procedure.

### CFU-F assay

The availability of fibroblast progenitors in fresh and thawed SVF aliquots (cryopreserved by methods C and D) was evaluated by CFU-F assay [9, 36, 37]. Cells were then resuspended and seeded at a final concentration of 5 × 10^2^ NC/cm^2^ in complete α-MEM. Complete α-MEM was obtained by adding 100 IU/ml penicillin, 100 μg/ml streptomycin (both from Sigma), and 10% vol/vol FBS (Lonza). Six-well plates (BD Biosciences, Bedford, MA, USA) were incubated at 37 °C under 5% CO_2_. After 24 h, the supernatant medium containing nonadherent cells was removed and discarded. After a single wash with phosphate-buffered saline (PBS; Lonza, Basel, Switzerland), fresh complete α-MEM containing 10% FBS was replaced on adherent cells. After 14 days, cells were washed with PBS, fixed with methanol, and stained by 5% vol/vol crystal violet (both from Sigma). Colonies were evaluated under an inverted phase-contrast microscope (Olympus CKX41, Olympus Italia Srl, Milano, Italy). Aggregates of more than 50 cells were considered as colonies.

### SVF differentiation potential assay

To assess the differentiation potential of freshly extracted or thawed SVF aliquots cryopreserved by methods C and D the following procedure was applied. SVF cells were resuspended in complete α-MEM containing 10% FBS and then cells were seeded at 5 × 10^4^ NC/cm^2^ in standard T25 tissue culture flasks (BD Biosciences). After 24 h, nonadherent cells were removed. After a single wash with PBS, α-MEM containing 10% FBS was replaced on adherent cells. After 7 days, cells were detached by trypsin-ethylenediaminetetraacetic acid (EDTA; TrypLe Select 10X, Life Technologies-Thermo Fisher Scientific, Waltham, MA, USA) and counted using a Cellometer Auto M10 (Nexcelom Bioscience, Lawrence, MA, USA). Adipogenic, chondrogenic, and osteogenic differentiations were achieved using StemMACS AdipoDiff, ChondroDiff, and OsteoDiff media (Miltenyi Biotec GmbH, Bergisch Gladbach, Germany) following the manufacturer’s instructions. After 21 days, adipocytes, chondrocytes (spheroids), and osteocytes were stained by Oil Red-O, Safranin-O, and Alizarin Red (Sigma), respectively. Osteogenic and adipogenic differentiation was analyzed by staining quantification in captured images using MATLAB® (Mathworks, Natick, MA, USA) software. After background subtraction, results were expressed as the percentage covered area. Chondrogenic differentiation was quantified measuring spheroid size taking advantage of Motic Images Plus 2.0 software®. Spheroid volume was calculated using the formula: (width^2^ × length × 3.14) / 6 [38].

### SRGF production

Platelet concentrates were obtained as previously published [25] with minor modifications. Briefly, samples of platelet concentrates were obtained from washouts of leukocyte depletion filters intended for disposal, taken from platelet apheresis collection kits (Haemonetics MCS+ System; Haemonetics, Signy-Centre, Switzerland) after donation from healthy donors. Under a sterile biological safety hood, the filtered apheretic product was transferred to a sterile tube (BD Biosciences). Platelet activation was performed by the addition of CaCl_2_ at a final concentration of 0.04 M (Monico SPA, Venice, Italy) and by incubation at 40 °C for approximately 60 min, i.e., until complete clot formation. Supernatants of centrifuged samples were stored at −80 °C until analysis. To obtain SRGF batches containing consistent amounts of growth factors we created pools of 16 single-donor SRGF products [26]. The obtained product was filtered through a 0.22-μm mesh filter (Merk Millipore, Darmstadt, Germany). Sterility tests were performed by standard procedures. Two separate batches were utilized in the present work.

### Ex-vivo expansion of ASC from thawed SVF cells

Thawed SVF cells were separately plated in standard T25 tissue culture flasks (BD Biosciences) with complete α-MEM medium plus 10% vol/vol FBS and in parallel with 5% vol/vol SRGF. SRGF was used at 5% as we previously demonstrated that further increasing the SRGF concentrations failed to confer any relevant advantages in terms of the ASC proliferation rate [28]. Cells, seeded at a cell density of 5 × 10^4^ NC/cm^2^, were allowed to adhere for 24 h. Nonadherent cells were then removed, and fresh medium was added after a single wash with PBS. Upon 80–90% confluence, cells (considered to be ASC) were detached by trypsin-EDTA. Resuspended cells were seeded (at passage (P)1 and at each following cell passage) at four different densities: 1 × 10^2^ cells/cm^2^, 1 × 10^3^ cells/cm^2^, 5 × 10^3^ cells/cm^2^, and 1 × 10^4^ cells/cm^2^. At each passage, the theoretical cell yield (TCY) was calculated.

The following equations were applied: TCY at P_i_ = n1_i_ × e[ln(2)/PDT_i_], where PDT_i_ = (tP_i_ – tP_i-1_) × 24/PDN at P_i_. (tP_i_ – tP_i-1_) × 24 is the time interval (in hours) between consecutive cell passages, and PDN at P_i_ = 3.32 × (log n2_i_ – log n1_i_). n1_i_ is the number of seeded cells and n2_i_ is the number or harvested cells at the selected passage (P_i_). When only part of the harvested cells were seeded, the following equation was applied: TCY at P_i_ = TCY at P_i–1_ × e[ln(2)/PDT_i_].

ASC expanded in α-MEM medium plus 10% vol/vol FBS or 5% SRGF were considered to be at low passages at P2–P3 [39]. ASC expanded in α-MEM medium plus 10% vol/vol FBS were considered to be at high passages when between P7 and P9 [39]. Otherwise, a posteriori considering the expansion rate of ASC expanded in α-MEM medium plus 5% vol/vol SRGF, such cells were considered to be at high passages when between P12 and P14.

### Morphometric analysis of expanded ASC

Images of high and low passage ASC expanded in α-MEM medium plus 10% vol/vol FBS or with 5% vol/vol SRGF were taken 24–48 h after seeding at 1 × 10^3^ cells/cm^2^ by phase-contrast microscopy (Olympus) and digital color camera (Motic). Morphometric analysis was performed taking advantage of Motic Images Plus 2.0 software® after appropriate calibration. At least 60 cells per captured image were manually analyzed. Measures of the cell major and minor axes as well as cell area were quantified using appropriate software tools following the manufacturer’s protocol.

### ASC differentiation potential assay

Adipogenic, chondrogenic, and osteogenic differentiation potential was assayed at high and low passages in ASC expanded by complete 10% FBS or 5% SRGF media at the plating density of 1 × 10^3^ cells/cm^2^. Cells were detached by trypsinization, and adipogenic, chondrogenic, and osteogenic differentiations were achieved utilizing StemMACS AdipoDiff, ChondroDiff, and OsteoDiff media (Miltenyi Biotec GmbH). After 21 days, osteocytes, adipocytes, and chondrocytes (spheroids) were stained by Oil Red-O, Safranin-O, and Alizarin Red (Sigma), respectively. Cell images were taken by inverted phase-contrast microscope (Olympus) and digital color camera (Motic). Osteogenic and adipogenic differentiation was analyzed by staining quantification in captured images using MATLAB® (Mathworks) software. After background subtraction, results were expressed as the percentage of covered area. Chondrogenic differentiation was quantified measuring spheroid size taking advantage of Motic Images Plus 2.0 software®. Spheroid volume was calculated using the formula: (width^2^ × length × 3.14)/6 [38].

### Cell transformation assay

ASC expanded until high passages in complete 10% FBS or 5% SRGF media at 1 × 10^3^ cells/cm^2^ were detached by trypsinization, and resuspended in serum-free α-MEM plus 100 IU/ml penicillin and 100 μg/ml streptomycin. A total of 4 × 10^3^ cells resuspended in 200 μl medium were further diluted with 1 ml of a methylcellulose-based medium (MethoCult H4230, StemCell Technologies, Vancouver, BC, Canada) [40]. After repeated resuspension by syringe (BD Biosciences) equipped with a blunt-end needle, cells were plated (in duplicate) in two wells of a 24-well plate (BD Biosciences). After 14 days, colony formation was checked by inverted microscope. Aggregates containing at least 50 round-shaped cells clearly distinguishable from underlying adherent cells were considered as colonies. HT1080 sarcoma cells (used as a positive control) were expanded in complete α-MEM plus 10% FBS, according to standard cell culture protocols. Images were taken by inverted phase-contrast microscope (Olympus) and digital color camera (Motic).

### Karyotype analysis

ASC expanded until high passages in complete 10% FBS or 5% SRGF media at 1 × 10^3^ cells/cm^2^ were detached by trypsinization. When a consistent fraction of proliferating round-shaped cells was evident on phase-contrast microscopy, karyotype analysis was performed according to standard procedures [41]. Captured images of metaphasic chromosomes were analyzed by CytoVision® software (Leica, Wetzlar, Germany). At least 20 metaphases were analyzed for each cell type.

### Statistics

Data are presented as mean ± SEM. Paired or unpaired Student’s *t* tests were applied to compare mean cell viability in the SVF product before freezing (Trypan blue exclusion test vs 7-AAD test; immediate vs delayed lipoaspirate manipulation; with vs without addition of red blood cell lysis solution). The impact of the different cryopreserving solutions on SVF cell viability upon thawing was analyzed by one-way analysis of variance (ANOVA) for independent samples. Impact of longer term cryostorage on SVF samples was analyzed by ANOVA for repeated measures. Tukey’s honestly different significance (HSD) with Bonferroni’s correction was chosen as a post-hoc test. Significance of the difference between means in a posteriori identified “High” and “Low” groups was tested by unpaired Student’s *t* test. Linearity of growth curves was tested calculating *R*^2^ as a measure of goodness of fit of linear regression. Differences between regression coefficients (slopes) of growth curves were tested by the Regression Model Analysis Test. Differences in cell morphology were analyzed by ANOVA for independent samples with interaction with Tukey’s HSD with Bonferroni’s correction as a post-hoc analysis.

## Results

### SVF cell characterization before freezing

**Fig. 2:**
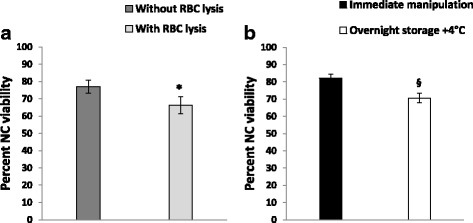
**a** Impact of red blood cell (RBC) lysis on percent nucleated cell (NC) viability measures in SVF. **b** Impact of overnight lipoaspirate storage at +4 °C on percent NC viability measured by Trypan blue dye exclusion test (without red blood cell lysis) in fresh SVF samples. *p < 0.01, vs without RBC lysis (Student’s *t* test for paired data); ^§^*p* < 0.05, vs immediate manipulation (Student’s *t* test for unpaired data)

The mean volume of lipoaspirates was 66.8 ± 4.7 ml. SVF mean cell yield evaluated at the end of the extraction protocol (before freezing) was 185.4 ± 19.3 × 10^3^ NC/ml lipoaspirate. We also evaluated cell viability by Trypan blue dye exclusion test in the fresh SVF and, as displayed in Fig. 2a, NC viability measured in a subset of fresh SVF aliquots treated by red blood cell lysis solution was significantly (*p* < 0.01) lowered when compared with matched untreated SVF aliquots (77.1 ± 3.9% vs 66.3 ± 4.9%). Figure 2b shows that the mean NC viability (measured without red blood cell lysis) in SVF extracted from lipoaspirates stored for 16–18 h at +4 °C was significantly (p < 0.01) decreased when compared with immediately processed samples (82.2 ± 1.8 vs 70.6 ± 2.1). Freshly isolated SVF samples were processed for cytofluorimetric analysis. The mean NC viability evaluated through 7-AAD was 72.2 ± 7.6%.

**Fig. 3:**
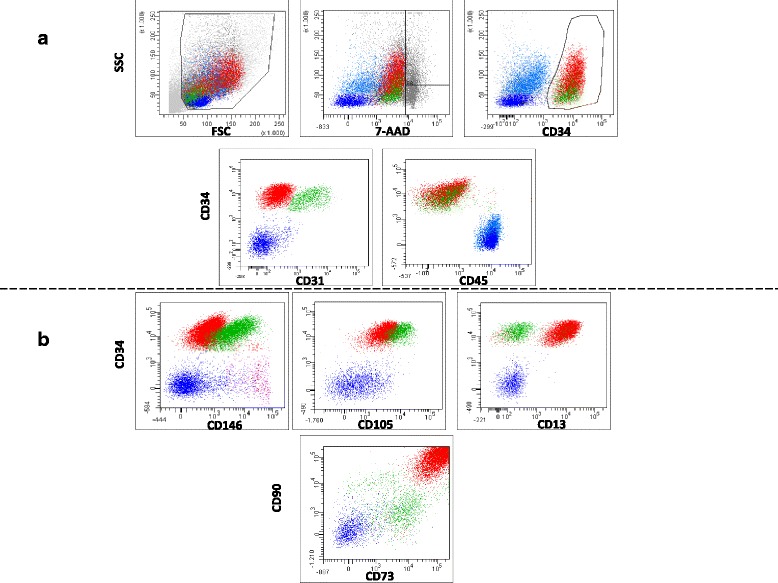
Representative flow cytometry immunophenotype analysis of SVF cells evaluated before freezing. **a** Gating strategy identifying three main populations in the SVF: CD34^+^CD31^−^CD45^−^ subset (ASC, red), CD45^−^CD34^+^CD31^+^ subset (EPC, green), and CD34^–^CD45^+^ subset (hematopoietic cells, blue). Dead cells (7AAD^+^) were excluded. **b** A detailed CD34^+^ cell characterization, showing expression of CD13, CD105, CD73, and CD90 in ASC and EPC. Pericytes were identified as CD34^−^CD45^−^CD31^−^CD146^+^ population (in violet). Lymphocytes are showed as reference (dark blue)

Immunophenotypic characterization was performed adopting a multicolor strategy that allowed identification of different vital cell populations. In particular, as shown in Fig. 3a, we identified in the CD34^+^CD45^−^ population (58.1 ± 7.6% of NC) a CD34^++^CD31^−^SSC^high^ subset (ASC, putative adipose-derived stromal cells; 58.8 ± 16.6% of CD34^+^ cells) and a CD34^+^CD31^+^SSC^low^ subset (EPC, putative endothelial progenitor cells; 43.2 ± 16.6% of CD34^+^ cells).

The phenotype of CD34^+^ cells, and in particular of ASC, was then characterized in detail with a large panel of antibodies, as reported in Table 1 (part A) and in part shown in Fig. 3b. ASC were brightly positive for CD90 and CD73, positive for CD13, CD44, CD10, and HLA I/ABC, dimly positive for CD105, CD29, CD166, CD106, and CD146, and negative for CD36, CD144, CD11c, CD11b, CD14, Glyα, and HLA II/DR.

**Table 1:** Expression level of surface markers analyzed in adipose tissue-derived stem cells (ASC) present in the stromal vascular fraction (SVF) and in expanded ASC

Markers	Part A		Part B	
	ASC in SVF		Expanded ASC	
	Fresh	Thawed	10% FBS	5% SRGF
CD34	++	++	–	–
CD45	–	–	–	–
CD31	–	–	–	–
CD90	++	++	++	++
CD73	++	++	++	++
CD13	+	+	++	++
CD105	+−	+−	++	++
CD44	+	+	+	+
CD29	+−	+−	++	++
CD166	+−	+−	++	++
CD10	+	+	+	+
HLA I, ABC	+	+	+−	+−
HLA II, DR	–	–	–	–
CD106	+−	+−	–	–
CD36	–	–	–	–
CD146	+−	+−	–	–
CD235	–	–	–	–
CD144	–	–	–	–
CD11b	–	–	–	–
CD11c	–	–	–	–
CD14	–	–	–	–

Part A reports the expression levels of selected markers in the 34^++^ 31^−^SSC^high^ ASC contained in fresh or thawed SVF samples

Part B reports the expression levels of the same markers in expanded ASC in the presence of 10% fetal bovine serum (FBS) or 5% supernatant rich in growth factors (SRGF) and alpha-minimal essential medium (α-MEM)

Expression levels are described as + and – considering unlabeled cells as reference (see also Fig. 3): ++, bright expression; +, positive expression; +−, dim expression

As shown in Fig. 3, when compared with ASC, the EPC subset CD34^+^CD31^+^SSC^low^ expressed CD90 and CD73 less brightly, but it was characterized by higher expression of CD105 and CD146. EPC cells were CD13^−^. When compared with ASC, the EPC population expressed higher levels of CD144, CD36 and HLA II/DR, CD44 and CD10, while the expression level of the remaining markers was not different from ASC (data not shown). Among the CD34^−^ cells, we distinguished a CD34^−^CD45^+^ cell population of putative hematopoietic origin (32.5 ± 14.6% of NC) and a CD31^−^CD45^−^CD146^+^ small subset considered as pericytes (Fig. 3b). Overnight lipoaspirate storage at +4 °C before SVF isolation did not affect the expression pattern of NC immunophenotype markers.

### Impact of freezing on SVF cells

**Fig. 4:**
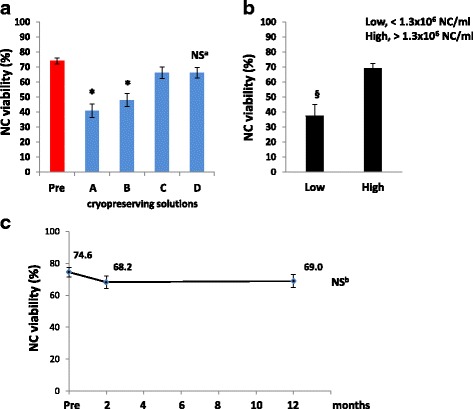
**a** Impact of different cryopreservation solutions on percent nucleated cell (NC) viability in thawed SVF products after 2 months of storage in liquid nitrogen. A, B, C and D are different cryopreservation solutions: solution A (10% Albital, 5% ACD-A, 10% DMSO, 75% saline solution), solution B (50% human serum, 5% Albital, 2.5% ACD-A, 10% DMSO, 32.5% saline solution), solution C (90% human serum, 10% DMSO), solution D (95% human serum, 5% DMSO). When compared with solutions C and D, the viability of NC stored for 2 months by solutions A and B was significantly lower. *p < 0.01, vs C and D; NS^a^, not significantly different vs C and vs Pre (one-way ANOVA for independent samples). **b** Impact of total NC concentration on SVF freezing on post-thaw cell viability. In the high NC concentration group (High), cell viability measured after thawing was significantly higher than in the low concentration (Low) group. ^§^p < 0.05, vs High Group (Student’s *t* test for unpaired data). **c** Impact of longer term cryostorage on SVF samples. NC viability measured after 1 year of freezing was not significantly different when compared with results obtained after 2 months storage. *p < 0.01, vs Pre (one-way ANOVA for independent samples). NS^b^, not significantly different vs 2 months (one-way ANOVA for repeated measures). Tukey’s honestly different significance with Bonferroni’s correction as post-hoc test

Cells were cryopreserved at the mean final concentration of 1.43 ± 0.7 × 10^6^ NC/ml in the different cryopreserving solutions (A, B, C, and D; see methods). The impact of cryopreservation on SVF mononuclear cell viability measured (upon thawing) by Trypan blue dye exclusion test is shown in Fig. 4a. Statistical analysis showed that post-thaw NC viability in SVF aliquots frozen for 2 months with both solutions A and B was significantly (*p* < 0.01) lower when compared to the viability measured in SVF before freezing. Otherwise, cell cryopreservation by solutions C and D fully prevented the loss of cell viability after freezing. In addition, no significant differences in post-thaw NC viability could be observed when comparing solution C with solution D. Since NC viability in SVF samples cryopreserved by solutions A and B was markedly lower (−34.6 ± 4.9%) when compared with samples cryopreserved by solutions C and D, solutions A and B were not considered as eligible for clinical applications. We then investigated the impact of NC concentration in the freezing mixture on post-thaw SVF cell viability. Cell viability data obtained in thawed SVF samples after 2 months of storage with solutions C and D were grouped together and a posteriori stratified according to NC concentration measured in each sample upon freezing (i.e., after resuspension in cryopreserving solution). Two groups were identified across a defined threshold of 1.3 × 10^6^ NC/ml: the group (*n* = 10) showing significantly higher NC concentration (1.95 ± 0.19 × 10^6^ NC/ml, *p* > 0.05) was defined as High, and the other group (*n* = 4) was defined as Low (0.52 ± 0.12 × 10^6^ NC/ml). As displayed in Fig. 4b, mean post-thaw cell viability measured in the High group (69.0 ± 3.2%) was significantly (*p* < 0.05) higher than in the Low group (37.5 ± 7.7%). In parallel, we aimed to evaluate thawed NC viability changes after a prolonged cryopreservation period. NC viability measured after 1 year of storage in solutions C and D was not significantly different when compared with viability results measured after 2 months of freezing (Fig. 4c). The phenotype evaluated after short- and long-term freezing (data not shown) was superimposable on that obtained in fresh samples (Table 1).

### Functional characterization of SVF cells

**Fig. 5:**
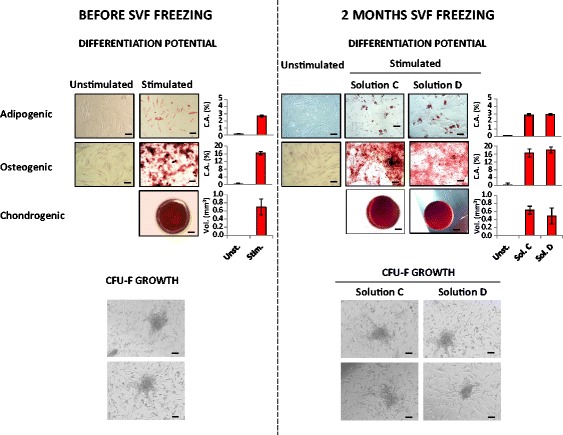
Representative images of osteogenic, adipogenic, and chondrogenic differentiation assays as well as colony forming unit-fibroblast (CFU-F) assays performed on stromal vascular fraction (SVF) cells before freezing or after 2 months of storage in the presence of cryopreservation solutions (Sol.) C and D. Differentiation was induced (Stim.) by the addition of commercially available osteogenic, adipogenic, and chondrogenic differentiation media to cells at passage P1. Unstimulated cells (Unst.; control) were cultured with 10% FBS medium. Within the chondrogenesis assay, spheroids failed to be obtained from unstimulated cells. Differentiated cells as well as unstimulated samples were stained with Alizarin Red, Oil Red-O, and Safranin-O to detect osteocytes, adipocytes, and chondrocytes, respectively. The differentiation degree was quantified by image analysis of cell staining (adipogenesis and osteogenesis) or by morphometric analysis of spheroids (chondrogenesis); results are reported in histograms. Sample storage in the presence of solutions C and D did not significantly affect cell differentiation potential. Scale bar = 100 μm. C.A., covered area; Vol., volume

Thawed SVF cells were seeded on a tissue culture plastic surface and adherent putative ASC were analyzed. As shown in Fig. 5, we demonstrated adipogenic, osteogenic, and chondrogenic differentiation of adherent ASC cells (P1) before SVF freezing and after cryopreservation with solutions C and D. Representative images of adipogenic, osteogenic, and chondrogenic differentiation assays are reported in Fig. 5; as further evidenced by analytical quantification, the two cryopreservation methods did not affect the cell differentiation potential. Representative images of CFU-F colonies obtained in fresh SVF samples as well as in SVF samples frozen by cryopreserving solutions C and D are displayed in Fig. 5.

### Ex vivo ASC expansion

**Fig. 6:**
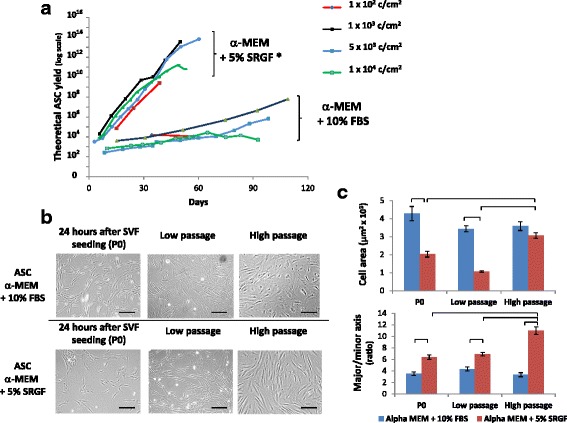
**a** Growth curves (logarithmic scale) of adipose tissue-derived stem cells (ASC) seeded at different cell densities (from 1 × 10^2^ to 1 × 10^4^ cells/cm^2^) in culture medium containing 10% fetal bovine serum (FBS) or 5% supernatant rich in growth factors (SRGF). The presence of SRGF in the cell culture induced a significantly higher growth rate when compared with FBS. **b** Plastic-adhering cells at P0 and after short-term (Low passage) or longer-term (High passage) expansion in the presence of 10% FBS or 5% SRGF in the cell culture medium. Scale bars = 100 μm. **c** Cell morphometric analysis. ASC expanded in 5% SRGF medium were smaller than those expanded in 10% FBS medium. The cell area of ASC cultured in 5% SRGF medium was greater at high passage when compared with P0 and low-passage cells. ASC expanded in 5% SRGF medium were more elongated than ASC expanded in 10% FBS medium during all culture phases. Linearity of growth curves was tested by calculating *R*^2^ as a measure of goodness of fit of linear regression. Differences between regression coefficients (slopes) of growth curves were tested by a Regression Model Analysis Test. *p < 0.01, vs FBS. Error bars of ASC growth curves could not be graphically reported in the diagram (logarithmic scale of *y* axis); the coefficient of variation regarding each plotted (mean) value of theoretical cell yield was below 10%. Connectors in **c** link significantly different means (*p* < 0.001, ANOVA for independent samples with interaction with Tukey’s HSD with Bonferroni’s correction as post-hoc analysis). MEM, minimum essential medium; SVF, stromal vascular fraction

**Fig. 7:**
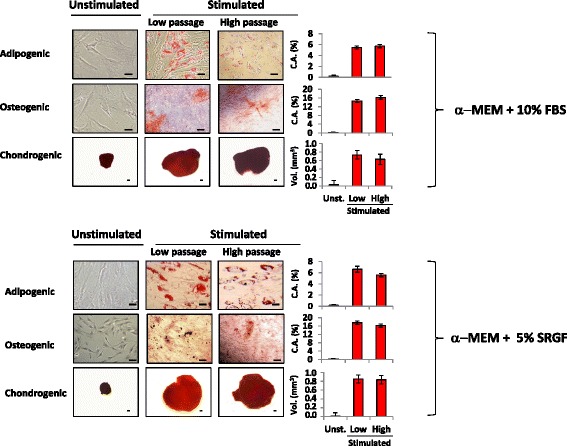
Representative images obtained from osteogenic, adipogenic, and chondrogenic differentiation assays performed on ASC after short-term or longer-term expansion at 1 × 10^3^ cells/cm^2^ in the presence of 10% fetal bovine serum (FBS) or 5% supernatant rich in growth factors (SRGF) in the cell culture medium. The differentiation degree was quantified by image analysis of cell staining (adipogenesis and osteogenesis) or by morphometric analysis of spheroids (chondrogenesis); results are reported in histograms. The differentiation potential was shown to be not significantly affected when comparing ASC expanded in 10% FBS and in 5% SRGF-containing media, both at high and low passages. Scale bar = 100 μm. C.A., covered Area; MEM, minimum essential medium; Vol., volume; Unst., unstimulated

**Fig. 8:**
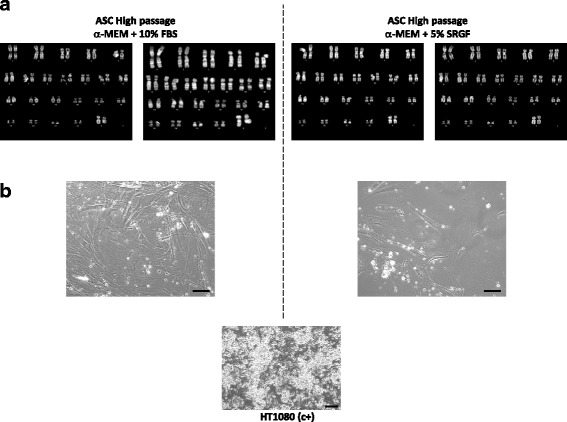
**a** Representative karyotypes of adipose tissue-derived stem cells (ASC) expanded at high passages in 10% fetal bovine serum (FBS)- or 5% supernatant rich in growth factors (SRGF)-containing medium. At least 20 metaphases were analyzed and no clonal or recurrent chromosomal alterations could be identified. **b** Displays images taken from colony formation assays in methylcellulose medium performed on high-passage ASC cultured in 5% SRGF- or 10% FBS-containing medium. ASC expanded utilizing both cell culture media failed to display colony formation. HT1080 fibrosarcoma cells were used as positive control (c+). Scale bar = 100 μm. MEM, minimum essential medium

We expanded ASC extracted from SVF thawed products comparing, at different cell seeding densities, the effect of media supplementation with 5% SRGF and 10% FBS on the ASC proliferation rate. Figure 6a shows, in a logarithmic scale, changes in TCY over time (days) measured by culturing cells in the presence of 5% SRGF or 10% FBS at different plating densities. When cultured in the presence of 10% FBS or 5% SRGF, ASC seeded at 1 × 10^3^ cells/cm^2^, 5 × 10^3^ cells/cm^2^, and 1 × 10^4^ cells/cm^2^ underwent several passages in culture without evidence of growth rate reduction. When compared with 10% FBS, 5% SRGF induced an overall higher ASC proliferation rate independent of cell seeding density (Fig. 6a). Considering the same cell culture medium, slopes of growth curves (linear, *R*^2^ = 0.98 ± 0.01) were not significantly affected by cell seeding densities at 1 × 10^3^ cells/cm^2^, 5 × 10^3^ cells/cm^2^, or 1 × 10^4^ cells/cm^2^. Otherwise, when seeded at 1 × 10^2^ cells/cm^2^ both in 10% FBS and 5% SRGF media, growth arrest rapidly occurred. In particular, ASC cultured in 10% FBS underwent growth arrest at P1 in ASC derived from 2 out of 5 patients, at P3 in 2 out of 5, and P6 in the last case. Similarly, when seeded at 1 × 10^2^ cells/cm^2^ in presence of 5% SRGF, growth arrest occurred at P1 in ASC derived from 1 out of 5 patients, at P4 in 3 cases, and at P6 in the last case. At the seeding density of 1 × 10^2^ cells/cm^2^, growth in isolated cell aggregates was frequently observed (data not shown). Figure 6b shows representative images of cultured ASC in 10% FBS- or in 5% SRGF-supplemented medium at P0, at low passages, and at high passages. Morphometric analysis of plastic-adherent ASC expanded in 5% SRGF or 10% FBS containing media is reported in Fig. 6c; independent of cell passage number, ASC expanded in 5% SRGF medium were smaller than ASC expanded in 10% FBS medium (*p* < 0.001). The cell culture method and the cell passage number significantly interacted (*p* = 0.002) to modulate adherent cell dimensions. Post-hoc analysis demonstrated that the cell area of ASC cultured in 5% SRGF medium was greater at high passages when compared with P0 and low passage cells. Still, independent of cell passage, ASC expanded in 5% SRGF medium were more elongated than ASC expanded in 10% FBS medium (*p* < 0.001). The effect of cell culture and cell passage number significantly interacted (*p* < 0.001) to modulate the adherent cell shape. Post-hoc statistical analysis demonstrated that, when compared with ASC grown in 10% FBS medium, ASC expanded in 5% SRGF medium maintained a greater axis ratio, especially at high passage. The immunophenotype of ASC expanded in 10% FBS or 5% SRGF medium was analyzed both at early and late passages. The panel of analyzed surface markers was substantially the same as that adopted to evaluate NC in SVF samples, including markers suggested by IFATS/ISCT [9]. As shown in Table 1 (part B), cells expressed CD90, CD73, CD13, CD105, CD29, and CD166 at high intensity, were positive for CD10, CD44, and HLA-ABC, and were negative for CD34, CD45, CD31, HLA-DR, CD106, CD36, CD146, CD235, CD144, CD11b, CD11c, and CD14. Moreover, CD13, CD105, CD29, and CD166 were overexpressed in expanded ASC when compared with nonexpanded ASC in SVF. CD34 expression was lost along with expansion, as expected. The expression level of the selected surface markers was stable from cell passage P3, and was maintained until late passages, with no substantial differences between cells grown with 10% FBS or 5% SRGF (Table 1, part B). Representative images of the differentiation potential of ASC expanded in 10% FBS or 5% SRGF at low and high cell passages are reported in Fig. 7. As also evidenced by analytical quantification, the adipogenic, osteogenic, and chondrogenic differentiation potential was shown to be not significantly affected when comparing ASC expanded in 10% FBS and in 5% SRGF containing media, both at high and low passages. Chromosome number and structure were analyzed in expanded ASC at high passage, and Fig. 8a shows examples of the obtained karyograms. In all analyzed samples, at least 20 metaphases were analyzed, and no clonal or recurrent chromosomal alterations could be identified.

Figure 8b shows representative images of colony formation assays in methylcellulose medium performed on high-passage ASC cultured in 5% SRGF- and 10% FBS-containing media. In all the performed assays, ASC colony formation on methylcellulose failed to be seen.

## Discussion

In the present study, we describe a method to extract SVF cells from lipoaspirates derived from breast cancer patients who underwent quadrantectomy or total mastectomy and reconstructive lipotransfer. A total of 19 lipoaspirates were processed to reliably set up and define the isolation protocol. Furthermore, we identified a safe method to cryopreserve and freeze SVF cells with minimal impact on cell viability or clonogenic and differentiation potential. Due to the limited amounts of extracted cells from each lipoaspirate, we could apply the different cryopreservation approaches only to subgroups of SVF samples. Moreover, we investigated the impact on the ASC proliferation rate, identity, differentiation potential, and cell stability mediated by SRGF, considered as a GMP-compliant medium additive for the expansion of ASC to obtain an ATMP. For practical and technical reasons, investigations regarding in vitro expanded ASC were limited to five SVF specimens as a starting product.

The mean quantity of NC extracted from lipoaspirates as well as the variability of the cell extraction yield (coefficient of variation, 42.5%) was in agreement with previous publications [3, 42]. Interindividual differences between lipoaspirate donors could explain the observed variability [3, 42]. To assay the SVF cell viability we utilized the Trypan blue dye exclusion manual test, as suggested by European Pharmacopeia [43]. Viability results published in previous papers were higher [3, 42] or comparable [5] when matched to our present data. Cell viability data were sufficiently consistent since the coefficient of variation between collected results was 13.1%. The presence of dead cells in fresh SVF products can be ascribed to mechanical adipose tissue disruption during the liposuction procedure, as well as to cell separation, washing, and concentration steps along with the isolation protocol [5]. In accordance with previous data [44], we showed that overnight storage of lipoaspirates at +4 °C exerts a mild detrimental effect on fresh SVF cell viability. Evaluation of cell viability is a quality control test within the manufacturing process and, thus, the biological specimen should at best represent the product condition as administered to the patient. We avoided red blood cells lysis, frequently used to facilitate evaluation of cell viability, as such a procedure is not normally performed on the final product aimed at patient administration. Due to extensive adipose tissue washing, the occurrence of residual red blood cells in the final product was minimized. Moreover, our results suggested that the lysis procedure could cause an underestimation of fresh SVF cell viability.

The characterization of the SVF cells was performed through a multiparametric immunophenotype analysis based on flow cytometry. Unfortunately, no unique single markers have been found so far; therefore, the analysis of a combination of markers is necessary to identify a cell subset sharing the same function and phenotypic signature. Results derived from the large panel of analyzed antigens were consistent with the minimal criteria proposed in the IFATS/ISCT position paper [9] and with previous reports [45–47]. In particular, as shown in Fig. 3 and Table 1, we identified a cell population CD34^++^CD31^−^CD45^−^, previously defined [9, 48] as ASC with mesenchymal phenotype, and a cell subset CD34^+^CD31^+^CD45^−^ that we can bona fide define as EPC. In addition, we could identify a CD34^−^CD31^−^CD45^−^CD146^+^ small population that can be putatively classified as pericytes [48]. The availability of such cell populations in fresh and thawed SVF confirms the possibility of applying this product in regenerative medicine applications since ASC, pericytes, and endothelial cells can synergistically cooperate to induce the formation of new blood vessels in an optimal regenerative microenvironment [2]. In this study, SVF aliquots were frozen using different cryopreservation solutions; NC viability was minimally affected in SVF samples cryopreserved with solutions containing pure serum and DMSO at both final concentrations of 5% and 10%. Thus, as previously demonstrated [3, 5], DMSO concentrations in pure serum can be reduced to 5% to correctly cryopreserve SVF cells. Under such conditions, cell viability in the frozen product was shown to be stable for at least 1 year. In addition, we have further demonstrated [3, 5] that SVF cells can be frozen in serum with 5% (and 10%) DMSO without affecting post-thaw CFU-F growth as well as ASC differentiation capacity. Reducing DMSO gives a significant advantage for clinical utilization of the thawed product since DMSO is known to be cytotoxic. Our approach can be considered as potentially compliant with GMP guidelines since animal-derived components were not used, and certified products or disposables were used for the manufacturing process. Moreover, we showed that post-thaw cell viability can be strongly reduced when freezing SVF at a cell concentration lower than 1.3 × 10^6^ NC/ml (putative threshold roughly corresponding to 1.0 × 10^6^ viable NC/ml). The definition of limited cell concentration for optimal freezing is crucially important for future clinical applications.

In the second part of this work, we aimed to optimize the ASC expansion protocol. ASC expansion is mandatory for cell therapy applications in humans. As an ATMP, expanded ASC must be produced in compliance with current GMP guidelines. FBS is progressively replaced in cell cultures by growth factors derived from human alternative sources [16] since xeno-carbohydrates and xeno-proteins may lead to undesired clinical effects [12–15]. Mesenchymal stem cells were previously expanded in vitro in a serum-free medium with a mixture of commercially available growth factors; nevertheless, under such conditions, the expression of selected surface markers was shown to be affected [49, 50]. Moreover, utilization of a coating substrate allowing cell adhesion is often required and the cost of media and reagents is considerably high. Utilization of human platelet-derived growth factors is compliant with GMP guidelines [17–20, 25]. In previous publications, growth factors were derived by repeated freeze and thaw cycles [18, 21–24]. Cells grown under such conditions showed morphology, as well as proliferation, immunomodulation, and differentiation potential, comparable with cells grown in FBS-containing media [18, 51, 52]. In this study, we used SRGF as standardized medium additive to stimulate ex-vivo ASC proliferation [25, 28]. Knowledge on the impact of such a medium additive on ASC physiology in cell culture is limited. Ancillary product standardization is suggested by GMP guidelines [21, 53] and, as we previously demonstrated, pooling together 16 single-donor products allowed a satisfactory batch-to-batch consistency [26]. Our results regarding cell growth kinetics demonstrated that SRGF dramatically increased the ASC proliferation rate when compared with FBS; this effect was demonstrated considering changes in cell yield at different cell passages. The expansion rate of bone marrow mesenchymal stem cells in the presence of growth factors derived from CaCl_2_-activated platelets was previously shown to be higher when compared with standard FBS [17, 28]. In a recent work, we demonstrated that, when compared with FBS and platelet lysate as medium additives, SRGF induced the highest proliferation rate also in bone marrow mesenchymal stem cells [54]. In this study, we tested ASC growth kinetics at different seeding densities and, independent of cell media composition, we showed that seeding cells at 1 × 10^3^ cells/cm^2^ can represent an optimal choice to expand ASC, reducing repeated exposures to trypsin while obtaining a satisfactory final cell yield. However, in a previous publication, ASC displayed changes in gene expression profiles in relation to cell seeding density: proliferation-related genes were highly expressed in cells expanded at low density, whereas genes regulating chemotaxis or differentiation properties were highly expressed in ASC expanded at high density (5 × 10^3^ cells/cm^2^) [55]. We demonstrated that ASC, rapidly expanded in the presence of 5% SRGF at 1 × 10^3^ cells/cm^2^, were characterized by a satisfactory capacity to differentiate into osteoblasts, chondrocytes, and adipocytes. Cell morphology was previously associated with proliferation rate and differentiation potential and, in particular, small and spindle-shaped cells were recognized as rapidly dividing cells, while bigger flatter ones were considered as slowly replicating cells [56, 57]. Considering our cell morphology analysis, we demonstrated that, when compared with FBS, the ASC expanded in SRGF-containing medium were smaller at early passages and generally more elongated. These results are in accordance with our data demonstrating a higher ASC proliferation rate in the presence of SRGF, even at extended cell passages.

To confirm the identity of ASC, a panel of surface markers was analyzed considering the ISCT recommendations [10]. The cell immunophenotype of ASC expanded in 10% FBS or 5% SRGF medium was analyzed both at low and high passages, and the obtained expression pattern of surface markers was substantially in line with previous reports [1, 9, 10]. Moreover, from the early passages, a pure and stable cell population sharing the same surface marker expression profile was detected for ASC expanded both in 10% FBS and 5% SRGF medium. Furthermore, the antigen expression level was not differently affected throughout the expansion process. Moreover, no difference was demonstrated when considering different cryopreserving solutions (namely, solutions C and D) used to store the SVF product (data not shown). As expected [1], when compared to nonexpanded ASC contained in the SVF, CD34 expression was blunted in expanded ASC and the expression level of CD13, CD105, CD29, and CD166 was increased (Table 1, part B). Under our experimental conditions, ASC did not show the expression of CD36; this marker was also reported to be negative in bone marrow mesenchymal stromal/stem cells [45, 58]. Within the process of expansion, ASC could potentially develop genetic instabilities [59], and chromosomal alterations may lead to apoptosis or cell death, and also to cell transformation [60]. In this study, we analyzed the cell karyotype and no genetic lesions in ASC expanded at high passages in the presence of both 5% SRGF and 10% FBS were seen. Transformed cells normally gain the capacity to grow under anchorage-independent conditions [61]. ASC expanded in both SRGF- and FCS-containing media failed to form cell colonies on methylcellulose [40]. In a previous work, ASC expanded in a xeno-free medium did not display features of tumor transformation despite a high proliferation rate [62]. Thus, considering our results, we can suggest that ASC expanded in 5% SRGF medium can be considered as genetically stable. Nevertheless, only further controlled clinical studies will shed light on the long-term tumor formation risk secondary to cell therapy treatments with expanded ASC.

## Conclusions

In conclusion, in this study we identified a safe and GMP-compliant protocol to extract, freeze, and thaw NC contained in SVF from adipose tissue. Freezing SVF aliquots at the appropriate cell concentration with a cryopreserving solution containing low (5%) DMSO concentration in pure serum failed to affect cell viability after short- and medium-term storage. The availability of an appropriate extraction, freezing, and thawing protocol is very important to manage the timing of product administration to patients. Afterwards, we provided a better description of the influence of SRGF as medium additive on the fundamental features of expanded ASC in vitro. Immunophenotype characterization and functional properties of such rapidly proliferating cells were in accordance with published guidelines [9, 10] and expanded ASC were not transformed. In this way, we defined a safe method to obtain, by a GMP-compliant protocol, expanded ASC from thawed SVF. This whole approach was set up in order to be easily “translated” within an authorized GMP manufacturing facility for the production of ATMPs.

## Abbreviations

ASC: Adipose-derived stromal/stem cells
ATMP: Advanced-Therapy Medicinal Product
CFU-F: Colony forming unit-fibroblasts
DMSO: Dimethylsulfoxide
FBS: Fetal bovine serum
GMP: Good Manufacturing Practice
MEM: Minimum essential medium
NC: Nucleated cells
PBS: Phosphate-buffered saline
SRGF: Supernatant rich in growth factors
SVF: Stromal vascular fraction
TCY: Theoretical cell yield
